# An Exponential Scale Mixture Model for Metatranscriptomics Data with Application to Inflammatory Bowel Disease

**DOI:** 10.64898/2026.05.15.725552

**Published:** 2026-05-15

**Authors:** Hyotae Kim, Li Ma

**Affiliations:** Biostatistics & Bioinformatics, Duke University; Department of Statistics and Data Science Institute, University of Chicago

**Keywords:** Scale mixture exponential modeling, Lomax distribution, Differential expression analysis, Metatranscriptomics

## Abstract

Metatranscriptomic (MTX) sequencing enables profiling of gene expression across microbial communities, providing a framework for linking genetic potential with functional activity. However, standard pipelines report normalized abundances rather than raw counts, limiting the use of count-based RNA-seq methods, while Gaussian-based alternatives rely on transformations and assumptions that are often poorly suited to MTX data. We propose a new modeling framework for differential expression analysis of MTX data, built on a scale mixture of exponential distributions, that incorporates DNA abundance to adjust for genomic potential, accommodates subject-specific random effects, treats zeros as left-censored, and employs a mixture prior to handle extreme sparsity. Applied to the IBDMDB multi-omics cohort, differential expression results vary substantially across models, including among Gaussian approaches with different pseudocount choices. Our approach identifies a distinct subset of candidate genes not detected by existing Gaussian methods; these may provide useful leads toward a novel understanding of transcriptomic patterns associated with dysbiosis in inflammatory bowel disease. Estimated dysbiosis effect directions are consistent between our model and Gaussian-based approaches, while effect sizes from our model tend to be larger in absolute value.

## 1 Introduction

While RNA-seq and scRNA-seq are dominant and well-established sequencing technologies for gene expression analysis, metatranscriptomic sequencing is rapidly gaining traction, as it can profile RNA from entire microbial communities; metatranscriptomic (MTX) analysis thereby enables investigations that connect community genetic potential with molecular activity (e.g., Franzosa et al. 2014, Bashiardes et al. 2016, Lloyd-Price et al. 2019).

To process metatranscriptomic sequencing data, several MTX pipelines—integrated collections of bioinformatics tools—have been developed. HUMAnN (Franzosa et al. 2018, Beghini et al. 2021) is one representative example; a comprehensive comparison of existing pipelines can be found in Shakya et al. (2019). These workflows typically produce transcript abundances normalized by gene length as RPK (reads per kilobase) and then further normalized by sequencing depth, reported as RPKM (reads per kilobase per million mapped reads) or CPM (copies per million), rather than as raw (i.e., unnormalized) counts. The absence of raw count output constrains potential modeling strategies, as count-based methods originally developed for RNA-seq and scRNA-seq are no longer applicable. Even when raw counts are available as intermediate outputs, additional information on the lengths of all identified genes is required, and no optimal normalization method for gene lengths exists for use within count-based models, further complicating their application.

Zhang et al. (2021) introduced Gaussian-based modeling methods for processed MTX abundance data that adjust for DNA gene copy number (also referred to as DNA abundance or metagenomic (MGX) abundance) to account for taxonomic and genomic potential variation across samples, a source of confounding in differential expression analysis. Similarly, other methods originally developed for RNA-seq but not count-based – for instance, zero-inflated beta regression (Peng et al. 2016), truncated Gaussian hurdle models (Finak et al. 2015), and linear models with weights (Law et al. 2014) – may be considered for MTX analysis with adjustment for DNA abundance.

We propose a new modeling framework based on a scale mixture of exponential distributions, in which the scale parameter is reparameterized as the product of a mean component and an auxiliary variable. The mean component incorporates DNA abundance, as in the Gaussian model discussed above, together with covariates of interest, including one for differential expression (DE) analysis. To account for repeated measurements from study participants, random effects can also be incorporated. An inverse-gamma prior is assigned to the auxiliary variable; marginalizing over this variable yields a Lomax model whose mean equals the mean component (hence the term mean component) and whose variance is expressed as a function of the inverse-gamma hyperparameter.

First, the proposed model addresses limitations of Gaussian-based approaches, including the need for data transformation with the addition of a pseudocount and the inadequacy of the normality assumption for the transformed data. Our model instead has support on the nonnegative real line, including zero, and features a decreasing density with a mode at zero, characteristics that make it well suited for MTX data, which are characterized by a predominance of zeros. Second, the Lomax distribution is heavy-tailed, allowing the model to accommodate nonzero observations, including those far from zero, even when zeros dominate the data. Third, we treat zeros as left-censored observations to account for technical zeros—values recorded as zero that may represent small positive quantities undetected due to pipeline artifacts—as well as biological zeros. Fourth, we replace the standard inverse-gamma prior with an inverse-gamma mixture prior for the auxiliary variable to account for excess zeros that may not be adequately captured even by the Lomax distribution in extremely sparse data, such as MTX measurements. Finally, posterior inference can be carried out using a general Gibbs sampler with closed-form full conditional distributions, yielding a tractable implementation.

The remainder of the paper is organized as follows. Section 2.1 describes the data structure produced by the HUMAnN pipeline, followed by a discussion of the limitations of an existing Gaussian-based method in Section 2.2. Sections 3.1 and 3.2 present our modeling framework and its key properties, respectively, while Section 3.3 completes the model construction by introducing strategies for handling zero measurements and their predominance in MTX data sets. The full hierarchical representation of the complete model and the prior specification are given in Section 4.1. Model implementation and posterior inference for differential expression are described in Sections 4.2 and 4.3. The simulation study (Section 5) and case study (Section 6), based on a publicly available multi-omics resource for inflammatory bowel disease, are used to evaluate the proposed approach and compare it with the existing Gaussian-based method. Finally, Section 7 concludes with a summary.

## 2 Motivating Example

### 2.1 MTX Data Description

In this paper, we analyze the Inflammatory Bowel Disease Multi’omics Database (IBDMDB; Lloyd-Price et al. 2019), available at https://ibdmdb.org. The resource provides both MGX and MTX data processed with the HUMAnN pipeline, along with associated metadata. These include community-level gene family abundances, each partitioned into contributions from individual organisms.

As an illustration of the data structure, Table 1 presents a subset of the MTX data for the gene family UniRef90_A0A015QIN6. Columns other than the first, which contains feature descriptions, correspond to samples, three of which are shown here and selected arbitrarily. The first row reports the aggregate gene family abundance, equal to the sum of the subsequent species-level abundances (prefixed by s), together with the corresponding genus-level information (prefixed by g). The last row, labeled “unclassified”, represents reads mapped to the gene family but lacking taxonomic assignment. All entries in the table are nonnegative real values representing CPM-scaled abundances.

**Figure 1:**
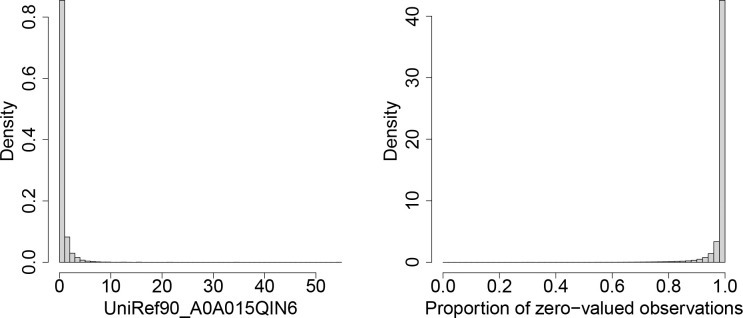
Histogram of transcript abundances for observations in the gene family UniRef90_A0A015QIN6 (left) and histogram of the proportion of zero-valued observations across gene families (right).

The MTX data consist of 817 samples and 1,088,367 gene families, each with associated taxa. For the gene family UniRef90_A0A015QIN6, approximately 55.45% of the species-level observations are zero-valued. The left panel of Figure 1 displays the distribution of the species-level observations for this gene, ranging from 0 to 54.6453, with the 75th percentile at 0.4846, indicating a highly right-skewed distribution.

The histogram in the right panel presents the distribution of the proportion of zero-valued observations across all gene families. The proportions range from 0 to 0.9988, with mean (median) values of 0.9854 (0.9963). The distribution is highly left-skewed, with even the 25th percentile at 0.9896.

Our approach, introduced in the following section, models the MTX data while incorporating the associated MGX data, provided in the same tabular format from the database, as a covariate for adjustment. As a preliminary step, we construct species-level paired abundance data for each gene family by removing the aggregated (first) and “unclassified” (last) rows and retaining only taxa common to both the MTX and MGX data sets.

Throughout the paper, we use the term “gene” to refer to an annotated gene family (UniRef90 cluster) as reported by the HUMAnN pipeline.

### 2.2 Limitations of Existing Gaussian Approaches

**Figure 2:**
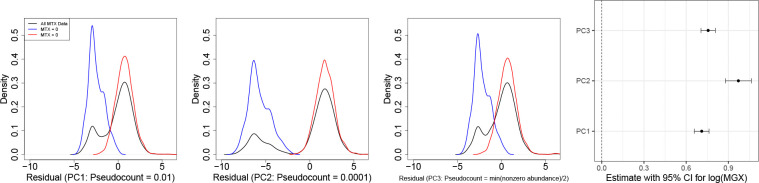
(UniRef90_A0A015QIN6) Density plots (via density () in R) of residuals from the linear model, lm (), under three pseudocount choices (0.01, 0.0001, half the minimum nonzero abundance) are shown in the first three panels, followed by the coefficient estimates with 95% confidence intervals for MGX on the log scale.

Figure 2 illustrates limitations of an existing Gaussian method proposed by Zhang et al. (2021). This approach requires a log transformation of the data, which in turn necessitates the addition of a pseudocount. The first three panels present the density of residuals from the Gaussian model, with MGX abundances (on the log scale) included as a covariate, under different pseudocount values. One choice (PC3), used in the reference paper, sets the pseudocount equal to one-half of the minimum nonzero value in the MTX and MGX data sets, respectively, to replace zero abundances in each data set. As suggested therein, sample pairs with both MTX and MGX equal to zero are excluded before model fitting; we discuss such filtering steps further in Section 3.1.

The resulting residual densities exhibit pronounced bimodality, with one mode corresponding to the density for nonzero MTX observations (MTX > 0) and the other to the density for observations with MTX = 0, which varies with the chosen pseudocount. Accordingly, the model’s normality assumption – which targets a symmetric, unimodal distribution – is inadequate for describing the data. Moreover, the coefficient estimates in the last panel demonstrate that the results also depend on the pseudocount choice, for which no universally accepted optimal value exists. Consequently, inference from such Gaussian-based differential expression models may be distorted by this choice. This distributional inadequacy and sensitivity to the pseudocount motivate the model development that follows.

## 3 Modeling Approach

In Section 3.1, we propose a base model and discuss its key properties in Section 3.2. This modeling framework underlies the final formulation presented in Section 3.3, where we also describe how zero abundances and their predominance are accommodated.

### 3.1 Base Modeling Framework

For a given gene, let $y_{ij}\in R^{+}$ denote the scaled abundance (e.g., RPKM or CPM) for taxon $j=1,\ldots ,J^{*}$ in sample $i=1,\ldots ,n^{*}$. Although the data can also be indexed by gene as $y_{ijs}$, with corresponding dimensions $J_{s}^{*}$ and $n_{s}^{*}$, where s denotes the gene, we suppress the index s for notational simplicity. The model proposed below provides a general framework applicable to each gene independently and can therefore be implemented in parallel across genes.

Let Lo(a,b) denote the Lomax distribution with shape a>0 and scale b>0, and mean b/(a−1) for a>1. Our base model is defined as

(1) $$y_{ij}|\alpha ,\beta ,\gamma ,\eta_{g(i)}\overset{ind.}{∼}Lo\left(\theta +1,\mu_{ij}\theta \right),\;\text{where}\;log\left(\mu_{ij}\right)=\alpha \;log\left(D_{ij}\right)+\beta s_{i}+\gamma^{\prime }X_{i}+\eta_{g(i)}.$$

Let Exp(λ) denote the exponential distribution with scale λ, which is also its mean. Equivalently, the model in (1) admits the following hierarchical representation with latent variable $U_{ij}$ as a scale mixture of exponential distributions:

(2) $$y_{ij}|\alpha ,\beta ,\gamma ,\eta_{g(i)},U_{ij}\;\overset{ind.}{∼}Exp\left(\lambda_{ij}\right),\;\lambda_{ij}=\mu_{ij}\times U_{ij},\;i=1,\ldots ,n,\;j=1,\ldots ,J_{i}\;\;log\left(\mu_{ij}\right)=\alpha \;log\left(D_{ij}\right)+\beta s_{i}+\gamma^{\prime }X_{i}+\eta_{g(i)}\;\;U_{ij}|\theta \overset{i.i.d}{∼}IG(\theta +1,\theta ),$$

where IG(a,b) denotes an inverse-gamma distribution with mean b/(a−1) and variance $b^{2}/[\left(a-1{)}^{2}(a-2\right)]$ for a>2. This representation is key to tractable inference, enabling closed-form posterior full conditionals for model parameters (detailed in Section 4.2). It also serves as the foundational framework for the complete model presented in Section 3.3, which we refer to as the exponential scale mixture (ESM) model. Model properties and a detailed derivation of the model equivalence are provided in Section 3.2.

Here, $D_{ij}$ represents the scaled DNA abundance, and $s_{i}$ is an observed covariate used in differential expression analysis. The vector $X_{i}={\left(1,x_{i1},\ldots ,x_{ip}\right)}^{\prime }$ contains an intercept and p additional covariates of interest, with associated coefficients $\gamma ={\left(\gamma_{0},\ldots ,\gamma_{p}\right)}^{\prime }$. In addition, $\eta_{g(i)}$ is a subject-specific random effect, where each subject g(i) may contribute multiple measurements (samples) in the longitudinal setting. $J_{i}$ and n denote the sample-specific number of genes and the number of patients, respectively, remaining after the filtering procedure described later, where $J_{i}\leq J^{*}$ and $n\leq n^{*}$. Because the remaining taxa may vary across samples after filtering, $J_{i}$ is sample-specific.

As in Zhang et al. (2021), we incorporate MGX abundance ($D_{ij}$) into the model to mitigate spurious differential expression findings, since DNA abundance can drive RNA abundance. Apparent high (or low) RNA abundance in a group may simply reflect underlying DNA abundance arising from taxonomic dominance (or rarity). For instance, changes in RNA abundance may result not from altered expression levels but from fewer gene copies due to a reduction in the abundance of taxa carrying the gene, as compositional shifts occur through the proliferation of other taxa (see Figure 1A of the reference paper for illustrative cases). By adjusting for DNA abundance, we better isolate expression changes attributable to the variable of interest.

MGX abundance can also be used for filtering. Zhang et al. (2021) describes a “semi-strict” (pre-)filtering procedure that removes samples when both MTX and MGX values are zero, representing a balanced approach between eliminating technical zeros and retaining sufficient samples, compared with two alternatives that are more permissive (“lenient”) or more stringent (“strict”). We adopt a strategy similar to the semi-strict filtering, in which we remove not only observations with both MTX and MGX values equal to zero but also those with zero MGX values alone, since apparent RNA expression without detectable gene copies is uncommon and may reflect technical artifacts.

Lastly, for the analyses in Sections 5 and 6, we use RNA and DNA abundances in CPM for $y_{ij}$ and $D_{ij}$, respectively. The grouping variable for differential expression analysis, $s_{i}$, termed dysbiosis, is defined as a binary indicator distinguishing dysbiotic samples (Croh’s disease [CD] or ulcerative colitis [UC]) from non-dysbiotic samples, that is, those deviating from healthy non-IBD MGX profiles (see Lloyd-Price et al. 2019, for details). We treat consent age and antibiotic use as covariates $x_{ik},\;k=1,\;2$, and participant ID as the subject index g(i). All data were obtained from the supplementary files *HMP2 Metadata* and *Dysbiosis Scores*, which accompany the MTX-MGX abundance data in the IBDMDB.

### 3.2 Key Model Properties

Under the modeling framework in (2), marginalization over the auxiliary variable $U_{ij}$ with its inverse-gamma prior yields a Lomax distribution in (1) (Proposition A.1, Supplementary Material), with mean $\mu_{ij}$ and variance $\mu_{ij}^{2}(\theta +1)/(\theta -1)$ for θ>1, and infinite variance for 0<θ≤1 (Proposition A.2, Supplementary Material).

The coefficient $\gamma_{k}$ for $x_{ik}$ is interpreted as the log fold change in the mean (as shown in Proposition A.3 of the Supplementary Material); analogous interpretations apply to α and β. The inverse-gamma hyperparameter θ, which modulates the variance, serves as a precision parameter.

**Figure 3:**
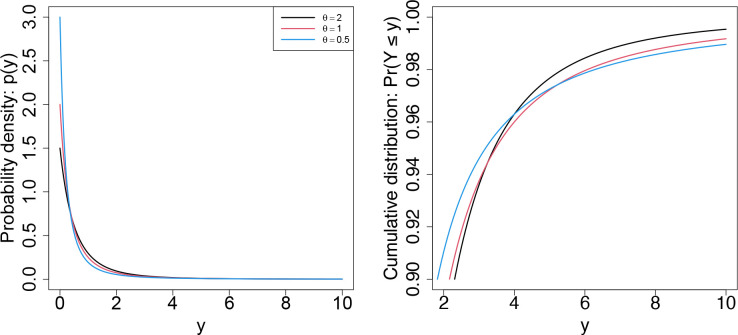
Lomax probability density and cumulative distribution functions under three different values of θ.

Figure 3 displays the probability density and cumulative distribution functions of the Lomax distribution, which is the sampling distribution for $y_{ij}$ under our base model. One notable characteristic is its support on the nonnegative real line, including zero. As such, the model accommodates zero-valued observations without transformation (i.e., without a pseudocount), although we discuss the treatment of zeros in light of the presence of technical zeros in the following section. Another important feature is the role of θ, which governs not only the variance but also the concentration of mass near zero and the heaviness of the tail.

Smaller values of θ induce greater concentration near zero while simultaneously producing heavier tails. Therefore, while the distribution shifts probability mass toward the origin to capture the high frequency of zero-valued observations, the heavy-tailed property ensures adequate accommodation of nonzero measurements. In the right panel, the smallest value of θ (0.5; shown in blue) exhibits the smallest 0.9 quantile while maintaining the heaviest tail, as evidenced by the smallest cumulative probability beyond approximately y=5.5. Formally, the model exhibits a polynomial tail with tail index (θ+1) (Proposition A.4, Supplementary Material), which is heavier than the tail of the log-normal distribution under the Gaussian model.

As a limiting property, the Lomax random variable converges to 0 in probability as θ→0. As θ→∞, it converges in distribution to an exponential distribution with scale parameter $\mu_{ij}$ (Proposition A.5, Supplementary Material).

### 3.3 Complete Model with Zero-Abundance Handling

While zero-valued observations can arise from the biological absence of an RNA transcript, they might also correspond to technical zeros resulting from the limit of quantification; for example, when transcript abundance is too low to be detected during preprocessing by a metatranscriptomic pipeline. This motivates our censoring approach, which treats a zero value as a censored realization of an underlying true expression level ${\tilde{y}}_{ij}$. Specifically, we observe $y_{ij}=0$ if ${\tilde{y}}_{ij}<C$, and $y_{ij}={\tilde{y}}_{ij}$ otherwise, for a given threshold C. Under this formulation, the contribution of the (i,j)-th MTX observation to the likelihood changes from the exponential density $exp\left(-y_{ij}/\lambda_{ij}\right)/\lambda_{ij}$ to

$${\left[exp\left(-y_{ij}/\lambda_{ij}\right)/\lambda_{ij}\right]}^{1\left(y_{ij}\neq 0\right)}{\left[Pr\left(y_{ij}<C\right)\right]}^{1\left(y_{ij}=0\right)},$$

where 1(x=z) denotes an indicator function that equals 1 if x=z and 0 otherwise. We adopt a data-augmentation strategy that introduces an auxiliary latent variable ${\tilde{y}}_{ij}$ for observations with $y_{ij}=0$, which ensures that posterior inference remains tractable (see Section 4.2). As an empirical choice, we set the threshold C to the minimum nonzero MTX abundance observed across all genes, which we interpret as the minimum detectable abundance in CPM.

As discussed in Section 3.2, the parameter θ controls the concentration of the data near zero: smaller values of θ correspond to greater concentration, heavier tails, and infinite variance for 0<θ<1. In our MTX data, many genes exhibit extreme sparsity, with zero-valued observations reaching up to 99.88% (median 99.63%). Such sparsity can drive θ toward zero during model fitting, presenting a challenge due to the model's limiting property: as θ→0, the distribution converges to a degenerate mass at zero. Consequently, although an infinitesimal estimate of θ implies infinite variance, it may still fail to adequately explain the data, as the implied density for $y_{ij}>0$ becomes vanishingly small. This issue persists even within the aforementioned censoring framework, where the model implicitly represents zeros via latent abundances ${\tilde{y}}_{ij}$, because these latent abundances, while nonzero, remain constrained to be small, with ${\tilde{y}}_{ij}<C$, where C denotes the minimum nonzero abundance across all genes. These considerations motivate a mixture formulation employing two distinct θ parameters, with the intent that one component accounts for nonzero values together with a portion of the latent abundances ${\tilde{y}}_{ij}$, while the other component captures the remaining latent abundances.

Specifically, we assume:

$$U_{ij}|\theta_{1},\theta_{2}\overset{i.i.d.}{∼}\rho IG\left(\theta_{1}+1,\theta_{1}\right)+(1-\rho )IG\left(\theta_{2}+1,\theta_{2}\right),\;i=1,\ldots ,n,\;j=1,\ldots ,J_{i}.$$

Section 4.1 provides a hierarchical representation of this complete model, incorporating a censoring mechanism for observations with y=0 and an inverse-gamma mixture for $U_{ij}$.

## 4 Posterior Inference

In Section 4.1, we describe the hierarchical formulation of the proposed model, followed by details of its implementation in Section 4.2. Section 4.3 presents a Bayesian approach to differential expression inference with adjustment for multiple testing.

### 4.1 Hierarchical Specification

Below we present the full hierarchical representation of the complete model, including all prior distributions. The participant identifier for sample i is denoted by ${PID}_{i}$, and $y=\left\{y_{ij}:i=1,\ldots ,n,j=1,\ldots ,J_{i}\right\}$. The model is given by

(3) $$\begin{array}{c} p(y|\alpha ,\beta ,\gamma ,\{\eta_{g(i)}\},\left\{U_{ij}\right\})\propto \prod_{i=1}^{n}\prod_{j=1}^{J_{i}}{\left[exp\left(-y_{ij}/\left(\mu_{ij}U_{ij}\right)\right)/\left(\mu_{ij}U_{ij}\right)\right]}^{1\left(y_{ij}\neq 0\right)}{\left[Pr\left(y_{ij}<C\right)\right]}^{1\left(y_{ij}=0\right)}\\ log\left(\mu_{ij}\right)=\alpha \;log\left(D_{ij}\right)+\beta s_{i}+\gamma^{\prime }X_{i}+\eta_{g(i)}\\ \alpha |\sigma^{2}∼\;N\left(0,\sigma^{2}\right)\\ \beta |\zeta ,\tau^{2}∼\left\{\begin{array}{ll} \delta_{0} & \text{if}\;\zeta =0\\ \;N\left(0,\tau^{2}\right) & \text{if}\;\zeta =1 \end{array}\right.\\ \zeta ∼Bern(\pi )\\ \tau ∼C^{+}(0,\psi )\Leftrightarrow \tau^{2}∼IG\left(1/2,\;1/b_{\tau^{2}}\right)\;\text{and}\;b_{\tau^{2}}∼IG\left(1/2,\;1/\psi^{2}\right)\\ \gamma_{k}|v_{k}^{2}\overset{\mathrm{i.i.d.}}{∼}N\left(0,v_{k}^{2}\right),\;k=0,\;1,\ldots ,p\\ \eta_{g(i)}|\phi^{2}\overset{\mathrm{i.i.d.}}{∼}N\left(0,\phi^{2}\right),\;g(i)\in \left\{{PID}_{i}:i=1,\ldots ,n\right\}\\ \sigma ,v_{k},\phi {\overset{\mathrm{i.i.d.}}{∼}C}^{+}(0,\psi )\\ U_{ij}|\theta_{1},\theta_{2},L_{ij}\overset{\mathrm{i.i.d.}}{∼}IG\left(\theta_{L_{ij}}+1,\theta_{L_{ij}}\right),\;i=1,\ldots ,n,\;j=1,\ldots ,J_{i}\\ Pr\left(L_{ij}=1\right)=\rho \;\text{and}\;Pr\left(L_{ij}=2\right)=1-\rho \\ \rho ∼Unif(0,\;1)\\ \theta_{1},\theta_{2}\overset{\mathrm{i.i.d.}}{∼}Exp(\xi ), \end{array}$$

where $N\left(\mu ,\sigma^{2}\right)$ is the normal distribution with mean μ and variance $\sigma^{2}$, and Bern(π) refers to the Bernoulli distribution with mean π.Unif(0,1) denotes an uniform distribution with support on the interval from 0 to 1. $\delta_{0}$ represents the Dirac delta function, which has zero density for all values except at 0. $C^{+}(0,\psi )$ is the half-Cauchy distribution with its density given by $p(y)=2/(\pi \psi )/\left(1+y^{2}/\psi^{2}\right)$ for y≥0.

For hypothesis testing of β in the DE analysis with respect to the variable $s_{i}$ (*dysbiosis*), we adopt a spike-and-slab prior. The formulation in (3) is a hierarchical representation of the prior with the latent variable ζ; after marginalizing over ζ, it reduces to the standard form $(1-\pi )\delta_{0}+\pi N\left(0,\tau^{2}\right)$. To reflect a balanced prior belief regarding zero-nonzero β, we set π=0.5.

All standard deviation parameters $\sigma ,\;v_{k}$, and ϕ, including τ (the standard deviation of the slab distribution), associated with the normal priors for the coefficients, are assigned half-Cauchy distributions. As a heavy-tailed prior, the half-Cauchy places more mass in the tails than exponentially tailed priors do; combined with its strong concentration near zero, it functions as an effective shrinkage prior, shrinking noise while preserving signals. We set ψ=10, chosen empirically based on sensitivity analyses over ψ=5,10,15, and 20. Specifically, we examined how β, the key parameter of interest in DE analysis, varies across these choices. Results of the sensitivity analysis for a simulation example are provided in Appendix B of the Supplementary Material, where the inference results, including TPR (true positive rate), FPR (false positive rate), and FDR (false discovery rate), are consistent across settings, with small discrepancies on the order of 0.01∼0.03 in the rates. Lastly, the half-Cauchy distribution prior allows for a hierarchical representation via inverse-gamma distributions for each corresponding variance (as shown in (3) for), which facilitates posterior sampling by yielding closed-form full conditional distributions for the variance parameters and their hyperparameters.

The weight ρ for the inverse-gamma mixture of $U_{ij}$ is assigned Unif(0,1), a noninformative prior for a probability parameter. For the parameters $\theta_{1}$ and $\theta_{2}$, which do not admit conjugate priors, we adopt independent exponential priors. In addition to the prior being parsimonious, with only one hyperparameter, the exponential density is monotonically decreasing and therefore favors smaller nonnegative values, which may be reasonable given the sparsity characteristic of MTX data. We conducted a sensitivity analysis with ξ=1 and 10 to assess the effect of ξ on estimates for β, including TPR, FPR, and FDR, and found the results to be robust in this regard (see Appendix B of the Supplementary Material), with a small discrepancy of 0.02 in TPR. We set ξ=10 for all subsequent analyses.

### 4.2 Model Implementation

Posterior inference is conducted using a Gibbs sampler, where closed-form full conditional distributions are available for most model parameters. To obtain these closed-form expressions, slice sampling with data augmentation plays a key role. First, the probability $Pr\left(y_{ij}<C\right)$ can be represented as $\int 1\left({\tilde{y}}_{ij}<C\right)\;exp\;\left(-{\tilde{y}}_{ij}/\left(\mu_{ij}U_{ij}\right)\right)/\left(\mu_{ij}U_{ij}\right)d{\tilde{y}}_{ij}$, where $\mu_{ij}=exp\{\alpha \;log\left(D_{ij}\right)+\beta s_{i}+\gamma^{\prime }X_{i}+\eta_{g(i)}\}$. Then, by introducing the latent variables $\tilde{y}=\left\{{\tilde{y}}_{ij}:y_{ij}=0,i=1,\ldots ,n,j=1,\ldots ,J_{i}\right\}$, the augmented likelihood is given by:

(4) $$p\left(y,\tilde{y}|\left\{\mu_{ij}\right\},\left\{U_{ij}\right\}\right)\propto \prod_{i=1}^{n}\prod_{j=1}^{J_{i}}{\left[\frac{exp\left(-y_{ij}/\left(\mu_{ij}U_{ij}\right)\right)}{\mu_{ij}U_{ij}}\right]}^{1\left(y_{ij}\neq 0\right)}{\left[\frac{1\left({\tilde{y}}_{ij}\;<\;C\right)exp\left(-{\tilde{y}}_{ij}/\left(\mu_{ij}U_{ij}\right)\right)}{\mu_{ij}U_{ij}}\right]}^{1\left(y_{ij}=0\right)}.$$

With additional latent variables $d_{ij}>0$, the exponential terms can be expressed as $exp(-\left.{\dot{y}}_{ij}/\left(\mu_{ij}U_{ij}\right)\right)=\int_{0}^{\infty }1\left(d_{ij}<exp\left(-{\dot{y}}_{ij}{\left(\mu_{ij}U_{ij}\right)}^{-1}\right)\right)dd_{ij}$, where ${\dot{y}}_{ij}=y_{ij}$ for observations with $y_{ij}\neq 0$ and ${\dot{y}}_{ij}={\tilde{y}}_{ij}$ for those with $y_{ij}=0$. Under the variable transformation $e_{ij}=-log\left(d_{ij}\right)$ with $e=\left\{e_{ij}:i=1,\ldots ,n,j=1,\ldots ,J_{i}\right\}$, the augmented likelihood is written as

(5) $$p\left(y,\tilde{y},e|\left\{\mu_{ij}\right\},\left\{U_{ij}\right\}\right)\propto \prod_{i=1}^{n}\prod_{j=1}^{J_{i}}1\left(e_{ij}>{\dot{y}}_{ij}{\left(\mu_{ij}U_{ij}\right)}^{-1}\right)\exp\left(-e_{ij}\right){\left(\mu_{ij}U_{ij}\right)}^{-1}{\left[1\left({\tilde{y}}_{ij}<C\right)\right]}^{1\left(y_{ij}=0\right)}.$$

The full conditional distribution of ${\tilde{y}}_{ij}$ for observations with $y_{ij}=0,\;i=1,\ldots ,n$ and $j=1,\ldots ,J_{i}$, is proportional to $1\left({\tilde{y}}_{ij}<C\right)exp\left(-{\tilde{y}}_{ij}/\left(\mu_{ij}U_{ij}\right)\right)$, as given in (4). Consequently, ${\tilde{y}}_{ij}$ is sampled from a truncated exponential distribution, $Exp\left(\mu_{ij}U_{ij}\right)$, with support restricted to ${\tilde{y}}_{ij}<C$.

The full conditional distribution for $e_{ij},\;i=1,\ldots ,n,\;j=1,\ldots ,J_{i}$, is given by $p\left(e_{ij}|\mu_{ij},U_{ij},y_{ij},{\tilde{y}}_{ij}\right)\propto \left(e_{ij}>{\dot{y}}_{ij}{\left(\mu_{ij}U_{ij}\right)}^{-1}\right)\;exp\left(-e_{ij}\right)$ from (5). Therefore, $e_{ij}$ follows a truncated exponential distribution, Exp(1) with support $e_{ij}>{\dot{y}}_{ij}{\left(\mu_{ij}U_{ij}\right)}^{-1}$. A posterior sample can be drawn by sampling from Exp(1) and adding ${\dot{y}}_{ij}{\left(\mu_{ij}U_{ij}\right)}^{-1}$ to the result.

The full conditional for α is derived from (5) as

$$\begin{array}{ll} p(\alpha |\beta ,\gamma ,\{\eta_{g(i)}\},y,\tilde{y},e) & \propto \prod_{i=1}^{n}\prod_{j=1}^{J_{i}}\left[1\left(e_{ij}>{\dot{y}}_{ij}\;exp\left(-\alpha \;log\left(D_{ij}\right)-\beta s_{i}-\gamma^{\prime }X_{i}-\eta_{g(i)}\right)U_{ij}^{-1}\right)\right.\\ & \times \;exp\left(-\alpha \;log\left(D_{ij}\right)\right)]\;exp\left(-\alpha^{2}/\left(2\sigma^{2}\right)\right)\\ & \propto \prod_{i=1}^{n}\prod_{j=1}^{J_{i}}\left[1\left(-log\left(e_{ij}U_{ij}/{\dot{y}}_{ij}\right)-\beta s_{i}-\gamma^{\prime }X_{i}-\eta_{g(i)}<\alpha \;log\left(D_{ij}\right)\right)\right]\\ & \times \;exp\left(-\sum_{i=1}^{n}\sum_{j=1}^{J_{i}}\alpha \;log\left(D_{ij}\right)-\alpha^{2}/\left(2\sigma^{2}\right)\right). \end{array}$$

Therefore, posterior samples of α are drawn from a truncated normal distribution, $N\left(-\sigma^{2}\sum_{i=1}^{n}\sum_{j=1}^{J_{i}}log\left(D_{ij}\right),\sigma^{2}\right)$, with lower and upper truncation bounds given by $\underset{\left\{i,j:log\left(D_{ij}\right)>0\right\}}{max}\left[\frac{-log\left(e_{ij}U_{ij}/{\dot{y}}_{ij}\right)-\beta s_{i}-\gamma^{\prime }X_{i}-\eta_{g(i)}}{log\left(D_{ij}\right)}\right]\;$ and $\underset{\left\{i,j:log\left(D_{ij}\right)<0\right\}}{min}\left[\frac{-log\left(e_{ij}U_{ij}/{\dot{y}}_{ij}\right)-\beta s_{i}-\gamma^{\prime }X_{i}-\eta_{g(i)}}{log\left(D_{ij}\right)}\right]$, respectively.

Similarly, each coefficient $\gamma_{k}$ associated with covariate $X_{ik}$ is sampled from a truncated normal distribution, $N\left(-v_{k}^{2}\sum_{i=1}^{n}\sum_{j=1}^{J_{i}}X_{ik},v_{k}^{2}\right)$, with lower and upper truncation bounds given by $\underset{\left\{i,j:X_{ik}>0\right\}}{max}\left[\frac{-log\left(e_{ij}U_{ij}/{\dot{y}}_{ij}\right)-\alpha \;log\left(D_{ij}\right)-\beta s_{i}-\sum_{q\neq k}\gamma_{q}X_{iq}-\eta_{g(i)}}{X_{ik}}\right]$ and $\underset{\left\{i,j:X_{ik}<0\right\}}{min}\left[\frac{-log\left(e_{ij}U_{ij}/{\dot{y}}_{ij}\right)-\alpha \;log\left(D_{ij}\right)-\beta s_{i}-\sum_{q\neq k}\gamma_{q}X_{iq}-\eta_{g(i)}}{X_{ik}}\right]$, respectively.

The random effect $\eta_{r}$, for r=1,…,R, where r indexes unique PIDs and R denotes their total number, is also drawn from a truncated normal distribution, $N\left(-\phi^{2}\sum_{i:g(i)=r}\sum_{j=1}^{J_{i}}1,\phi^{2}\right)$, with a lower truncation bound given by $\underset{\left\{i,j:g(i)=r\right\}}{max}\left[-\log\left(\frac{e_{ij}U_{ij}}{{\dot{y}}_{ij}}\right)-\alpha \;log\left(D_{ij}\right)-\beta s_{i}-\gamma^{\prime }X_{i}\right]$. The coefficient β for the DE analysis, assigned a spike-and-slab prior, is updated primarily according to ζ: when ζ=0,β=0; when ζ=1,β follows a truncated normal distribution, $N(-\tau^{2}\sum_{i=1}^{n}\sum_{j=1}^{J_{i}}s_{i},\tau^{2})$, with lower and upper bounds $\underset{\left\{i,j:s_{i}>0\right\}}{max}\left[\frac{-log\left(e_{ij}U_{ij}/{\dot{y}}_{ij}\right)-\alpha \;log\left(D_{ij}\right)-\gamma^{\prime }X_{i}-\eta_{g(i)}}{s_{i}}\right]$ and $\underset{\left\{i,j:s_{i}<0\right\}}{min}\left[\frac{-log\left(e_{ij}U_{ij}/{\dot{y}}_{ij}\right)-\alpha \;log\left(D_{ij}\right)-\gamma^{\prime }X_{i}-\eta_{g(i)}}{s_{i}}\right]$.

Regarding the update of ζ, we first integrate out β from the joint posterior distribution $p\left(y,\dot{y},e|\left\{\mu_{ij}\right\},\left\{U_{ij}\right\}\right)p(\beta )p(\zeta )$. Otherwise, ζ is deterministically defined by : if β=0, then ζ=0; if β≠0, then ζ=1. This leads to the full conditional distribution of ζ in the form ${\left[(1-\pi )h_{0}\right]}^{1(\zeta =0)}{\left[\pi h_{1}\right]}^{1(\zeta =1)}$, where $h_{0}=\prod_{i=1}^{n}\prod_{j=1}^{J_{i}}1(e_{ij}>{\dot{y}}_{ij}exp-\alpha \;log\left(D_{ij}\right)-\gamma^{\prime }X_{i}-\eta_{g(i)})U_{ij}^{-1})$ and $h_{1}=exp\left(\tau^{2}{\left(\sum_{i=1}^{n}\sum_{j=1}^{J_{i}}s_{i}\right)}^{2}/2\right)\int_{LB}^{UB}N(\beta |-\tau^{2}\sum_{i=1}^{n}\sum_{j=1}^{J_{i}}s_{i},\tau^{2})d\beta$. The integration bounds are given by $LB=\underset{\left\{i,j:s_{i}>0\right\}}{max}\left[\frac{-log\left(e_{ij}U_{ij}/{\dot{y}}_{ij}\right)-\alpha \;log\left(D_{ij}\right)-\gamma^{\prime }X_{i}-\eta_{g(i)}}{s_{i}}\right]$ and $UB=\underset{\left\{i,j:s_{i}<0\right\}}{min}\left[\frac{-log\left(e_{ij}U_{ij}/{\dot{y}}_{ij}\right)-\alpha \;log\left(D_{ij}\right)-\gamma^{\prime }X_{i}-\eta_{g(i)}}{s_{i}}\right]$. Consequently, a posterior sample of ζ can be drawn from a Bernoulli distribution with success probability $\pi h_{1}/((1-\pi )h_{0}+\pi h_{1})$.

Using the inverse-gamma representation of the half-Cauchy distribution, the variance parameters are sampled from updated inverse-gamma distributions. Specifically, for $\left(\sigma^{2},b_{\sigma^{2}}\right)$, the full conditionals are $IG\left(1/2+1/2,\alpha^{2}/2+1/b_{\sigma^{2}}\right)$ and $IG\left(1/2+1/2,1/\sigma^{2}+1/a^{2}\right)$, respectively. Similarly, for $\left(v_{k}^{2},b_{v_{k}^{2}}\right)$, the corresponding full conditionals are $IG(1/2+1/2,\gamma_{k}^{2}/2+1/b_{v_{k}^{2}})$ and $IG\left(1/2+1/2,1/v_{k}^{2}+1/a^{2}\right)$. The parameter $\tau^{2}$ depends on the latent variable ζ. When ζ=1 at a given MCMC iteration, $\tau^{2}$ is sampled from its inverse-gamma full conditional distribution, $IG\left(1/2+1/2,\beta^{2}/2+1/b_{\tau^{2}}\right)$; otherwise, it is sampled from its prior distribution. The auxiliary parameter $b_{\tau^{2}}$ is sampled from $IG\left(1/2+1/2,1/\tau^{2}+1/a^{2}\right)$. Finally, the variance parameter $\phi^{2}$ of the random effect is sampled from $IG(R/2+1/2,\sum_{r=1}^{R}\eta_{r}^{2}/2+1/b_{\phi^{2}})$, with $b_{\phi^{2}}$ sampled from $IG\left(1/2+1/2,1/\phi^{2}+1/a^{2}\right)$.

The posterior sampling of $U_{ij}$ is based on the likelihood (4) combined with its conjugate inverse-gamma prior, yielding $p(U_{ij}|\theta_{1},\theta_{2},L_{ij},\mu_{ij},y_{ij},{\tilde{y}}_{ij})\propto U_{ij}^{-1}exp(-{\dot{y}}_{ij}\mu_{ij}^{-1}U_{ij}^{-1})U_{ij}^{-\theta_{L_{ij}}-2}exp(-\theta_{L_{ij}}U_{ij}^{-1})$. Consequently, $U_{ij}$ follows an updated inverse-gamma distribution, $IG(\theta_{L_{ij}}+2,{\dot{y}}_{ij}\mu_{ij}^{-1}+\theta_{L_{ij}})$.

The mixture allocation indicator $L_{ij}$ is sampled from a discrete distribution with updated probabilities given by $Pr\left(L_{ij}=1\right)\propto \rho IG\left(U_{ij}|\theta_{1}+1,\theta_{1}\right)$ and $Pr\left(L_{ij}=2\right)\propto (1-\rho )IG\left(U_{ij}|\theta_{2}+\right.1,\theta_{2}$), where IG(x∣a,b) denotes the density function of an inverse gamma distribution IG(a,b).

Parameter ρ is updated using a beta distribution with parameters $n_{1}+1$ and $n_{2}+1$, where $n_{1}$ and $n_{2}$ denote the numbers of observations with $L_{ij}=1$ and $L_{ij}=2$, respectively.

The precision parameters $\theta_{1}$ and $\theta_{2}$ are the only variables that lack closed-form full conditional distributions; the Metropolis-Hastings algorithm is employed for their posterior sampling.

### 4.3 Posterior Inference for Differential Expression

Under the hierarchical formulation in (3), ζ=1 indicates a nonzero β. Posterior inference for testing β is conducted through the latent variable ζ, specifically using the posterior inclusion probability (PIP), defined as the posterior mean of ζ. The null hypothesis β=0 is rejected if the PIP exceeds a prespecified threshold, such as 0.95 or 0.99 (analogous to significance levels of 0.05 or 0.01 in classical hypothesis testing), and is retained otherwise. Since DE analysis generally involves testing multiple genes simultaneously, conducting independent tests with a common PIP threshold may lead to an inflation of false positives. This necessitates a Bayesian counterpart to multiple-testing adjustments, such as the control of the familywise error rate (FWER) or the false discovery rate (FDR). To this end, we identify a set of genes while bounding the expected proportion of false discoveries at a prespecified level, following the approach of Newton et al. (2004). Specifically, we rank genes in ascending order of 1 – PIP and truncate the list to control the FDR. Formally, all genes $s\in S_{m}$ are declared differentially expressed if $\sum_{s\in S_{m}}\left(1-{PIP}_{s}\right)/m\leq \alpha$, where α denotes the target FDR level, $S_{m}$ is the set of the first m genes with the largest PIP values, and m is the largest integer satisfying the inequality. The subsequent results in Sections 5 and 6 are based on this FDR-adjusted decision rule at a target level of α=0.05.

## 5 Simulation Study

We conducted a simulation study using data generated from IBDMDB, for which 1000 genes were arbitrarily selected according to the following criteria. We retained genes such that the number of species shared between the corresponding MTX and MGX data was at least two. We further restrict to genes for which, after MGX-based filtering, the two binary variables (dysbiosis and antibiotic use) are non-identical, with each variable having two categories represented by more than five observations, thereby avoiding perfect collinearity and model degeneracy and yielding more stable estimates. At the sample level, we excluded observations with missing metadata for any of the covariates, resulting in a final data set of 784 samples from the original 817 samples.

Although the dysbiosis variable may still be used, we generated an alternative binary variable to serve as the DE indicator in case one group is too rare. Specifically, we created a binary variable s by splitting observations evenly within both the zero and nonzero MTX measurements. We then multiplied MTX measurements in group s=1 by a positive signal strength. This construction induces a positive association for observations with s=1, where the coefficient β represents the logarithm of the signal strength. Conversely, a negative association was induced by dividing the MTX measurements for observations with s=1 by the same positive signal (alternatively, the same effect could be obtained by multiplying the measurements for observations with s=0). In generating s, we performed the splitting separately within the zero and nonzero subsets rather than across the full set of observations. This stratified approach promotes comparable baseline abundances between groups prior to MTX perturbation. Otherwise, if one group is dominated by zero values and contains too few nonzero measurements, the abundances in that group may not be comparable to those in the other group, potentially leading to false positives and distortion of the intended signal strength.

For the simulation study, we randomly designated 400 genes (40% of the total 1000 genes) for signal injection, which we refer to as the truly DE genes. To generate positive signals, the MTX abundances of 200 genes were multiplied by a factor of 10; for the other 200 genes, abundances were divided by 10 to create negative signals.

We undertook a comparative analysis with the Gaussian model (GM) of Zhang et al. (2021), fitted using lmer () in the lmerTest package, under several pseudocount choices, to demonstrate the relative performance of our exponential scale mixture model. Both frameworks incorporate multiple testing corrections to control the false discovery rate (FDR): specifically, we applied the Bayesian approach detailed in Section 4.3 for the ESM model and the Benjamini-Hochberg procedure for the Gaussian alternatives.

**Figure 4:**
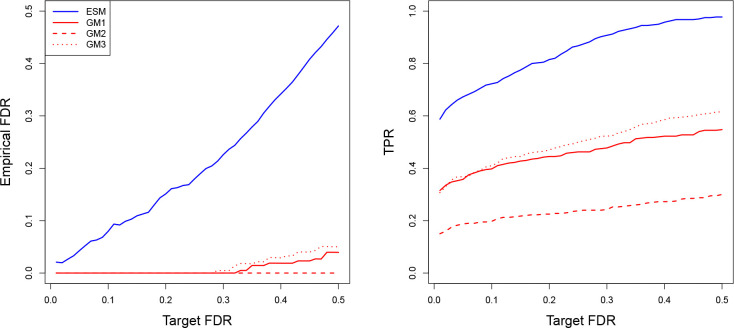
Calibration plot of empirical FDR versus target FDR level (α) on the left and power plot of TPR versus α on the right. ESM: exponential scale mixture model; GM#: Gaussian model with pseudocount PC#, where PC1 = 0.01, PC2 = 0.0001, and PC3 = half the minimum nonzero abundance.

Figure 4 shows the calibration and power plots, displaying the empirical FDR and TPR as the target FDR level α varies. The results indicate that our model provides substantially better FDR calibration than the Gaussian models, which are overly conservative and yield empirical FDR values of zero at commonly used target levels (0.05, 0.1, and 0.2). Moreover, for a given target level, the Gaussian models yield substantially lower TPRs than our proposed model.

Table 2 reports the adjusted TPR at matched empirical FDR levels. Even after accounting for the conservative nature of the Gaussian models – by allowing them to match the empirical FDR of our model using higher target levels – our model consistently outperforms the existing methods. The “NA” value for GM2 indicates that it cannot achieve the specified empirical FDR even when the target level is increased to 0.99.

**Figure 5:**
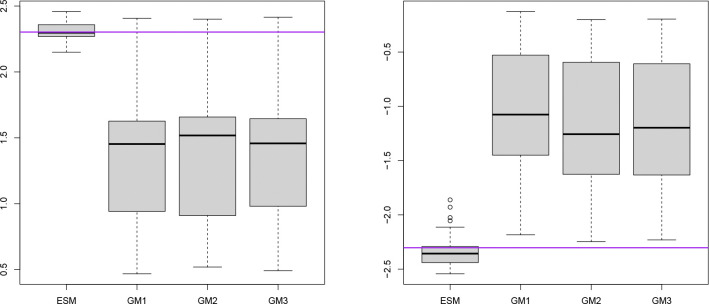
Box plots of positive (left) and negative (right) signal strength estimates for genes identified as differentially expressed by all models. Purple lines denote the true signal values.

Figure 5 presents the distributions of signal strength estimates, with true values log(10) for positive associations and log(1/10) for negative associations, across genes commonly identified by all models. The results reveal systematic bias in the GMs. The root mean squared errors (RMSEs) are (0.15,1.34,1.23,1.25) for (ESM,GM1,GM2,GM3), respectively. Our model demonstrates substantially better performance than the GMs. Among the GMs, GM2 (which yields the lowest adjusted TPR) shows the smallest discrepancy.

Additional results for an alternative signal strength and number of genes with injected signals are provided in Appendix C.

## 6 Case Study

As a key application, we conducted a DE analysis with respect to the dysbiosis variable for genes in IBDMDB. The results are based on 3000 genes randomly selected according to the criteria described in Section 5; we restrict attention to genes with at least one nonzero MTX measurement in each dysbiosis group so that log-fold change estimates are well defined. The analysis is based on 784 complete samples after excluding those with any missing metadata.

The model variables $\left\{s_{i},X_{i},g(i)\right\}$ for i=1,…,784 are summarized as follows. For dysbiosis status, 123 observations were classified as “ES” and 661 as “NO”. Age had a mean of 28.18 years, with first and third quartiles of 14 and 42, respectively. For antibiotic use, 65 observations were “YES” and 719 were “NO”. In total, the data set included 106 unique participant identifiers. An MGX-based filtering step was applied in which the sample-taxon pair (i,j) was excluded whenever $D_{ij}=0$.

**Figure 6:**
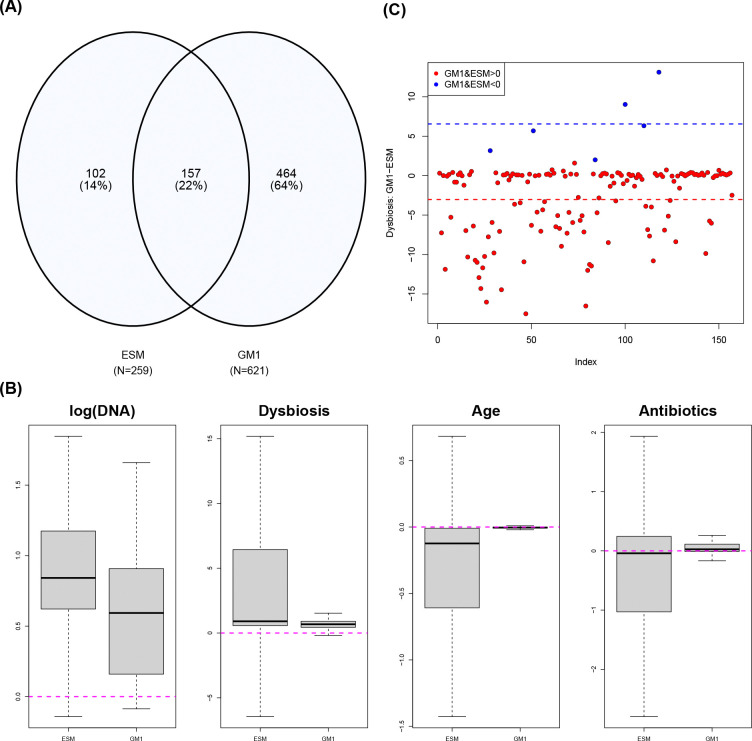
(A) Venn diagram of genes identified as differentially expressed with respect to the dysbiosis variable under ESM and GM1, with N denoting the number of identified genes. (B) Boxplots (with outliers omitted) of effect sizes for log(DNA), dysbiosis, age, and antibiotic use (i.e., posterior means of $\alpha ,\;\beta ,\;\gamma_{1}$, and $\gamma_{2}$) for the 157 commonly identified genes. (C) Pairwise differences in the dysbiosis effect sizes among the common genes across models.

A comparative analysis between our model and a Gaussian alternative (GM1 with pseudocount 10^−2^) is presented in Figure 6, illustrating how the two approaches can differ in a real-data analysis. The results are based on multiple testing adjustment using the procedure in Section 4.3 for ESM and the Benjamini-Hochberg procedure for GM1, with α=0.05. Panel (A) shows a Venn diagram of genes identified as significant for the dysbiosis covariate by each model. The number of identified genes varies substantially. Since the true number of differentially expressed genes is unknown, it is difficult to determine which model performs better; GM1 identifies substantially more genes as significant, but the number of identified genes decreases as the pseudocount decreases (454 genes for pseudocount 10^−4^ and 391 genes for pseudocount 10^−8^), demonstrating sensitivity to the choice of pseudocount. Notably, 102 out of 259 genes identified by ESM are exclusive to that model. Although the biological relevance of these ESM-specific genes remains to be determined, their protein names, effect sizes, and posterior inclusion probabilities are provided in Table A5 of the Supplementary Material for further investigation. Protein nomenclature was obtained from the UniProt database, and only genes with available database entries are included in the table.

We next examine the 157 genes commonly identified by both models. Panel (B) presents boxplots of the effect sizes for log(DNA), dysbiosis, age, and antibiotic use, revealing pronounced discrepancies not only in central tendency but also in variability. Panel (C) focuses on the dysbiosis effect sizes, our primary variable of interest. Red points correspond to genes with positive dysbiosis effect estimates, whereas blue points correspond to genes with negative dysbiosis effect estimates. ESM and GM1 agree on the sign of the effect for all the genes. However, ESM tends to produce stronger effects, yielding larger β values for positive effects and smaller β values for negative effects. Consequently, the means (dashed lines) of the red and blue points are shifted farther away from zero toward the negative and positive directions, respectively. This greater magnitude of absolute effect sizes may contribute to the larger variability observed for ESM in Panel (B).

Lastly, we conducted an additional comparative analysis of the three Gaussian models with different pseudocounts (GM1, GM2, and GM3), demonstrating pseudocount sensitivity not only in the identification of differentially expressed genes but also in the estimated effect sizes (see Figure A3 of the Supplementary Material).

## 7 Conclusion

Metatranscriptomic data are highly sparse; in the IBDMDB used in our application, the proportion of zeros is as high as 0.9896 even at the 25th percentile across genes. We have developed a novel modeling method – a scale mixture of exponential distributions – for differential expression analysis of such sparse data. This approach is motivated by the limitations of existing Gaussian-based methods, specifically distributional inadequacy and an inherent sensitivity to the choice of pseudocount, and naturally addresses these issues.

Our base modeling framework can be viewed as a hierarchical representation of a Lomax model in which the regression coefficients admit an interpretation as log fold changes in the mean, and the parameter θ governs the concentration of mass near zero as well as tail behavior and variance. Small θ yields greater concentration near zero (accommodating a large number of zeros), together with a heavier tail and infinite variance when 0<θ≤1 (allowing occasional large nonzero observations). However, the base model alone may be insufficient for extremely sparse data because the Lomax distribution converges to a degenerate point mass at zero as θ→0, implying that the Lomax density for nonzero values becomes negligible when θ is estimated to be very small. To overcome this limitation, we have employed a two-component mixture formulation with parameters $\theta_{1}$ and $\theta_{2}$, in which one component accounts for nonzero values and a subset of zeros, while the other captures the remaining excess zeros. We have further introduced a zero-handling mechanism based on a censoring formulation to account for both biological and technical zeros. Finally, posterior inference is tractable via a general Gibbs sampler with closed-form full conditional distributions, providing a practical advantage for implementation.

We illustrate our model and compare it with the existing Gaussian method using simulation data based on IBDMDB. The results show that our proposed model achieves better calibration, higher power, larger TPRs at matched empirical FDRs, and greater accuracy in signal strength estimation.

We further apply the methods to the IBDMDB multi-omics data. The DE analysis reveals substantial discrepancies across models in the number of identified genes, even among Gaussian models with different pseudocount choices. Our framework identifies a subset of genes not detected by the Gaussian-based alternative; their biological relevance remains to be determined, but they may provide useful leads for future investigation of transcriptomic patterns associated with dysbiosis in IBD patients. For dysbiosis effect estimation, all models yield consistent directions of effect, while our approach tends to produce larger absolute effect sizes.

## Supplementary Material

Supplement 1

## Figures and Tables

**Table 1:** Subset of MTX data showing transcript abundance for one gene family UniRef90_A0A015QIN6 across three samples (columns).

Gene Family	CSM5FZ3T	CSM5FZ46	CSM5FZ4G

UniRef90_A0A015QIN6	56.1085	5.34637	5.04212
UniRef90_A0A015QIN6|g_Bacteroides.s_Bacteroides_fragilis	34.9527	4.45395	0
UniRef90_A0A015QIN6|g_Bacteroides.s_Bacteroides_ovatus	0	0	1.67376
UniRef90_A0A015QIN6|g_Bacteroides.s_Bacteroides_uniformis	0	0	1.75071
UniRef90_A0A015QIN6|g_Bacteroides.s_Bacteroides_vulgatus	21.1558	0.89242	0
UniRef90_A0A015QIN6|g_Parabacteroides.s_Parabacteroides_distasonis	0	0	1.61765
UniRef90_A0A015QIN6|unclassified	0	0	0

**Table 2:** TPR (power) as a function of empirical FDR, showing the power-FDR trade-off across competing methods.

Empirical FDR	TPR			
	ESM	GM1	GM2	GM3

FDR ≈ 0.05	0.68	0.58	0.35	0.62
FDR ≈ 0.10	0.77	0.67	0.39	0.71
FDR ≈ 0.20	0.90	0.81	NA	0.87
