# Supplementary material for: An Exponential Scale Mixture Model for Metatranscriptomics Data with Application to Inflammatory Bowel Disease
Source: bioRxiv. 2026 May 15:2026.05.15.725552. Preprint. [Version 1] doi: 10.64898/2026.05.15.725552 (PMC13192739; doi:10.64898/2026.05.15.725552)
Supplement: Supplement 1 [file media-1.pdf]

Supplementary Material for “An Exponential Scale Mixture Model for Metatranscriptomics Data with Application to Inflammatory Bowel Disease”

Appendix A Proofs of Propositions

Proposition A.1 (Marginalization). *Suppose* $U_{ij} \sim \mathrm{IG}(\theta+1, \theta)$ *and* $Y_{ij} \mid \mu_{ij}, U_{ij} \sim \mathrm{Exp}(\mu_{ij} U_{ij})$. *Then marginally*

$$Y_{ij} \sim \mathrm{Lo}(\theta+1, \mu_{ij}\theta).$$

Proof. The density marginalized over U_{ij} is

$$\begin{aligned} p(y_{ij}|\mu_{ij}, \theta) &= \int_0^\infty (\mu_{ij}U_{ij})^{-1} \exp\Big(- y_{ij}(\mu_{ij}U_{ij})^{-1}\Big) \frac{\theta^{\theta+1}}{\Gamma(\theta+1)} U_{ij}^{-\theta-2} \exp(-\theta/U_{ij}) dU_{ij} \\ &= \int_0^\infty \frac{\mu_{ij}^{-1}\theta^{\theta+1}}{\Gamma(\theta+1)} \frac{\Gamma(\theta+2)}{(\theta + y_{ij}\mu_{ij}^{-1})} \mathrm{IG}\Big(U_{ij}|(\theta+2), (\theta + y_{ij}\mu_{ij}^{-1})\Big) dU_{ij} \\ &= \frac{(\theta+1)}{\mu_{ij}\theta}\Big(1 + \frac{y_{ij}}{\mu_{ij}\theta}\Big)^{\Big((\theta+1)+1\Big)}, \end{aligned}$$

where $\mathrm{IG}(y|a, b)$ denotes the density of an inverse-gamma distribution $\mathrm{IG}(a, b)$. The resulting marginal density corresponds to that of the Lomax distribution $\mathrm{Lo}(\theta+1, \mu_{ij}\theta)$. □

Proposition A.2 (Mean and variance). *For* $Y_{ij} \sim \mathrm{Lo}(\theta+1, \mu_{ij}\theta)$,

$$\mathrm{E}[Y_{ij}] = \mu_{ij}, \quad \theta > 0, \quad \mathrm{Var}(Y_{ij}) = \begin{cases} \mu_{ij}^2(\theta+1)/(\theta-1), & \theta > 1, \\ \infty, & 0 < \theta \le 1. \end{cases}$$

Proof. The moment generating function (MGF) of a Lomax random variable $Y \sim \mathrm{Lo}(a, b)$ is

$$M_Y(t) = a\exp(-bt)(-bt)^a\Gamma(-a, -bt),$$

where $\Gamma(-a, -bt)$ is the upper incomplete gamma function. Using the identity

$$\Gamma(-a, -bt) = \Gamma(-a) - \gamma(-a, -bt) = \Gamma(-a) + \sum_{k=0}^{\infty} \frac{(-bt)^{k-a}(-1)^k}{k!(a-k)},$$

where $\gamma(\cdot, \cdot)$ denotes the lower incomplete gamma function, we obtain

$$M_Y(t) = a\exp(-bt)\left[(-bt)^a\Gamma(-a) + \sum_{k=0}^{\infty} \frac{(-bt)^k(-1)^k}{k!(a-k)}\right].$$

Differentiating $M_Y(t)$ with respect to t gives

$$M'_Y(t) = a\exp(-bt)(-b)\left[(-bt)^a\Gamma(-a) + \sum_{k=0}^{\infty}\frac{(-bt)^k(-1)^k}{k!(a-k)}\right]$$
$$+ a\exp(-bt)\left[a(-bt)^{a-1}(-b)\Gamma(-a) + \sum_{k=1}^{\infty}\frac{k(-bt)^{k-1}(-b)(-1)^k}{k!(a-k)}\right].$$

The second derivative is

$$M''_Y(t) = a\exp(-bt)b^2\left[(-bt)^a\Gamma(-a) + \sum_{k=0}^{\infty}\frac{(-bt)^k(-1)^k}{k!(a-k)}\right]$$
$$+ 2a\exp(-bt)(-b)\left[a(-bt)^{a-1}(-b)\Gamma(-a) + \sum_{k=1}^{\infty}\frac{k(-bt)^{k-1}(-b)(-1)^k}{k!(a-k)}\right]$$
$$+ a\exp(-bt)\left[a(a-1)(-bt)^{a-2}b^2\Gamma(-a) + \sum_{k=2}^{\infty}\frac{k(k-1)(-bt)^{k-2}b^2(-1)^k}{k!(a-k)}\right].$$

Thus, the first and second moments are given by $\mathrm{E}(Y) = M'_Y(0) = b/(a-1)$ for $a > 1$ and $\mathrm{E}(Y^2) = M''_Y(0) = b^2(1 - 2a/(a-1) + a/(a-2))$ for $a > 2$. Consequently, the variance is $ab^2/[(a-1)^2(a-2)]$ for $a > 2$ and diverges to infinity for $1 < a \leq 2$ (undefined for $a \leq 1$). Substituting $a = \theta + 1$ and $b = \mu_{ij}\theta$ yields $\mathrm{E}[Y_{ij}] = \mu_{ij}$ and $\mathrm{Var}(Y_{ij}) = \mu_{ij}^2(\theta+1)/(\theta-1)$, which is finite iff $\theta > 1$. □

Proposition A.3 (Coefficient interpretability)**.** *Under the model* $\mu_{ij} = \exp\Big(\alpha\log(D_{ij}) + \beta s_i + \sum_{k=0}^{p}\gamma_k x_{ik} + \eta_{g(i)}\Big)$*, each coefficient* γ_k *represents the log fold-change in the mean for a one-unit increase in covariate* x_k*, holding all other terms constant.*

Proof. Let $\mu_{ij}(x_{ik})$ denote the mean μ_{ij} evaluated at a specific value of x_{ik}, with all other covariates held fixed. Following Proposition A.2,

$$\mu_{ij}(x_{ik}) = \exp\Big(\alpha\log(D_{ij}) + \beta s_i + \gamma_{-k}^{\top}(X_i)_{-k} + \gamma_k x_{ik} + \eta_{g(i)}\Big),$$

where $(X_i)_{-k}$ denotes the covariate vector excluding x_{ik}, and γ_{-k} is the corresponding coefficient vector excluding γ_k. Comparing two values x_{ik} and x'_{ik}, we obtain

$$\frac{\mu_{ij}(x_{ik})}{\mu_{ij}(x'_{ik})} = \exp\Big(\gamma_k(x_{ik} - x'_{ik})\Big).$$

Because the model uses an exponential mean structure (i.e., a log link), multiplicative effects on the mean scale become additive on the log scale, making γ_k interpretable as a log fold-change. $\square$

Proposition A.4 (Tail behavior)**.** *For $Y_{ij} \sim \text{Lo}(\theta+1, \mu_{ij}\theta)$, the distribution is regularly varying with tail index $(\theta+1)$ and is therefore heavy-tailed.*

Proof. The survival function is $\Pr(Y_{ij} > y) = \left(1 + \frac{y}{\mu_{ij}\theta}\right)^{-(\theta+1)} = y^{-(\theta+1)}L(y)$, where $L(y) = (1/y + 1/\mu_{ij}\theta)^{-(\theta+1)}$. Since $L(ty)/L(y) \to 1$ for all $t > 0$ as $y \to \infty$, $L(y)$ is slowly varying, and therefore the Lomax distribution is regularly varying with tail index $(\theta+1)$ $\square$

Proposition A.5 (Limiting behavior in θ)**.** *Let $Y_{ij}^{(\theta)} \sim \text{Lo}(\theta+1, \mu_{ij}\theta)$.*

1. *As $\theta \to \infty$, $Y_{ij}^{(\theta)} \to \text{Exp}(\mu_{ij})$ in distribution.*
2. *As $\theta \to 0^+$, $Y_{ij}^{(\theta)} \to 0$ in probability.*

Proof. (1) The cumulative distribution function of $Y_{ij}^{(\theta)}$ is

$$\Pr(Y_{ij}^{(\theta)} < a) = 1 - \left(1 + a/(\mu_{ij}\theta)\right)^{-(\theta+1)}.$$

Then

$$\lim_{\theta\to\infty} \Pr(Y_{ij}^{(\theta)} < a) = 1 - \left[\lim_{\theta\to\infty}\left(1 + a/(\mu_{ij}\theta)\right)^{\theta+1}\right]^{-1} = 1 - \left[\exp(a/\mu_{ij}) \times 1\right]^{-1} = 1 - \exp(-a/\mu_{ij}),$$

which is the cumulative distribution function of an exponential distribution with scale parameter μ_{ij}. Hence, $Y_{ij}^{(\theta)} \to \text{Exp}(\mu_{ij})$ in distribution.

(2) For every $\epsilon > 0$,

$$\Pr(Y_{ij}^{(\theta)} > \epsilon) = \left(1 + \epsilon/(\mu_{ij}\theta)\right)^{-(\theta+1)}.$$

As $\theta \to 0^+$, we have $(\theta+1) \to 1^+$ and $\epsilon/(\mu_{ij}\theta) \to \infty$, so $\Pr(Y_{ij}^{(\theta)} > \epsilon) \to 0$. By definition, $Y_{ij}^{(\theta)} \to 0$ in probability. $\square$

Appendix B Sensitivity Analysis

	$\psi = 5$	$\psi = 10$	$\psi = 15$	$\psi = 20$
TPR/FPR/FDR	0.83/0.03/0.06	0.81/0.03/0.05	0.80/0.03/0.06	0.82/0.03/0.05

Table A1: TPR, FPR, and FDR across values of ψ.

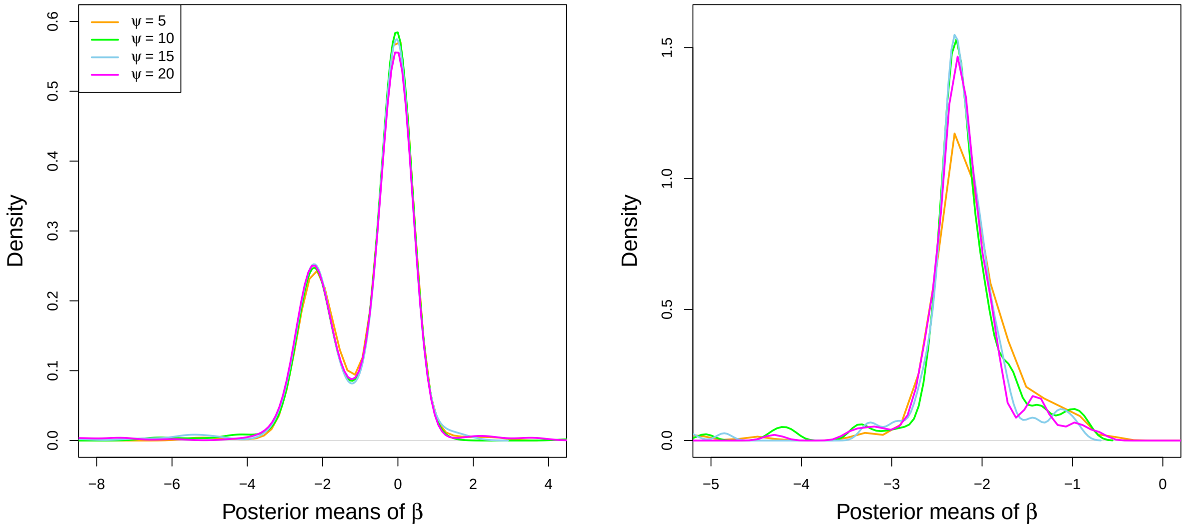

Figure A1: Densities (estimated using the `density` function in R) of the posterior means of β for all 500 genes (left) and for genes identified as DE from models with different values of ψ (right).

We conducted sensitivity analyses to assess the impact of the hyperparameters ψ and ξ. As in the simulation study in the main paper (Section 5), we first generated IBDMDB-based synthetic data for the 500 selected genes. For each gene, we created a new grouping variable s, assigning a value of 1 to half of the zero MTX observations and half of the nonzero observations. Then, for a random subset of 200 (40%) genes, we induced a negative signal by multiplying MTX abundances by a factor of 10 for observations with $s = 0$.

We first considered four values of ψ (5, 10, 15, and 20) and assessed sensitivity with respect

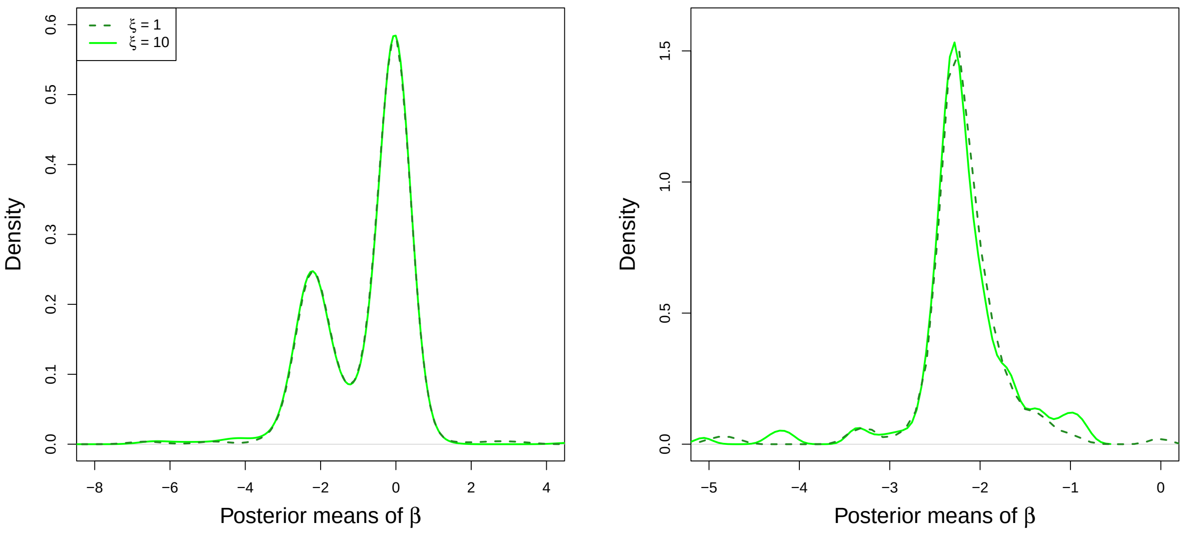

Figure A2: Densities of the posterior means of β for all 500 genes (left) and for genes identified as DE (right) under $\xi = 1$ and 10.

to β, the parameter of primary interest in DE analysis. Figure A1 displays (estimated) densities of the posterior means of β for all 500 genes and for subsets of genes identified as DE from models fitted with ψ fixed at each value. The density based on all 500 genes indicates that the model is robust to the choice of ψ. The right panel for the DE subsets also shows robustness, although minor discrepancies are present; for example, the curve for $\psi = 5$ exhibits a slight deviation near the mode relative to the others. Table A1 reports the corresponding TPR, FPR, and FDR values, which are nearly identical across specifications, with differences on the order of $0.01 \sim 0.03$.

	$\xi = 1$	$\xi = 10$
TPR/FPR/FDR	0.79/0.03/0.05	0.81/0.03/0.05

Table A2: TPR, FPR, and FDR across values of ξ.

Similarly, we evaluated sensitivity to the choice of $\xi = 1, 10$ with respect to the key

parameter β. Figure A2 shows densities of the posterior means of β, and Table A2 reports the corresponding TPR, FPR, and FDR, indicating robustness to the choice of ξ.

Appendix C Additional Results for Simulation Study

Table A3 presents the TPRs at specified empirical FDR levels for each method. Our model (ESM) consistently outperforms the Gaussian-based methods across all FDR thresholds. In several cases, the Gaussian methods yield NA values because they fail to reach the specified empirical FDR even when the target FDR increases to 0.99, indicating overly conservative behavior.

	Empirical FDR	TPR			
		ESM	GM1	GM2	GM3
Scenario 1	FDR ≈ 0.05	0.58	0.45	NA	0.51
	FDR ≈ 0.10	0.68	0.52	NA	0.60
	FDR ≈ 0.20	0.83	NA	NA	0.77
Scenario 2	FDR ≈ 0.05	0.65	0.57	NA	0.60
	FDR ≈ 0.10	0.68	0.62	NA	0.63
	FDR ≈ 0.20	0.78	NA	NA	NA
Scenario 3	FDR ≈ 0.05	0.53	0.41	NA	0.46
	FDR ≈ 0.10	0.59	NA	NA	0.50
	FDR ≈ 0.20	0.68	NA	NA	NA

Table A3: TPR (power) as a function of empirical FDR, showing the power–FDR trade-off across competing methods. Scenario 1: (signal strength, percentage of truly DE genes) $(5, 40\%)$; Scenario 2: $(10, 20\%)$; Scenario 3: $(5, 20\%)$.

Table A4 reports the RMSEs of signal strength estimates under each model for genes commonly identified by all models. In all scenarios, ESM consistently outperforms the Gaussian method, yielding substantially smaller RMSEs.

	ESM	GM1	GM2	GM3
Scenario 1	0.11	0.81	0.74	0.75
Scenario 2	0.15	1.44	1.34	1.34
Scenario 3	0.08	0.78	0.70	0.71

Table A4: RMSE of signal strength estimates for each model.

Appendix D Additional Result for Case Study

Gene Family	Protein Name (UniProtKB)	PIP	Effect Size
UniRef90_A0A081U0V8	FecR family protein	0.887	5.869
UniRef90_A0A0M6WAS1	Distal rod protein (Flagellar basal body rod protein FlgG)	0.858	1.798
UniRef90_A0A0P0F6D8	Uncharacterized protein	0.988	1.018
UniRef90_A0A139JXD7	Cyclodeaminase/cyclohydrolase family protein (Formimidoyltetrahydrofolate cyclodeaminase (EC 3.5.4.9, EC 4.3.1.4))	0.895	0.652
UniRef90_A0A139KER2	Uncharacterized protein	0.921	2.750
UniRef90_A0A173Y691	Copper-sensitive operon repressor	0.981	9.290
UniRef90_A0A174FBF5	Glucosamine-6-phosphate deaminase (EC 3.5.99.6) (Glucosamine-6-phosphate isomerase)	0.849	8.362
UniRef90_A0A174NDI4	Methylated-DNA–protein-cysteine methyltransferase (EC 2.1.1.63) (6-O-methylguanine-DNA methyltransferase) (MGMT) (O-6-methylguanine-DNA-alkyltransferase)	0.849	0.516
UniRef90_A0A174UXE2	Transposase and inactivated derivatives	0.975	16.010
UniRef90_A8RLB8	Sodium:solute symporter	1.000	12.872
UniRef90_B0MXN4	Transporter, SSS family	0.855	4.939
UniRef90_B0MYD0	Methyltransferase domain protein	0.830	4.320
UniRef90_C0FVH6	Uncharacterized protein	0.917	5.182
UniRef90_D1JNU2	Cysteine-rich CWC family protein	0.875	1.091
UniRef90_D4N102	Acyl-CoA dehydrogenase (EC 1.3.8.1) (Butyryl-CoA dehydrogenase (EC 1.3.99.2))	0.871	-8.122
UniRef90_G4Q739	Small ribosomal subunit protein uS17	0.922	10.334
UniRef90_I9PWB6	SPP1 family phage portal protein	0.835	-1.228
UniRef90_P0C652	Insertion element IS1 protein InsA	1.000	11.101
UniRef90_Q5LFT6	Aminomethyltransferase (EC 2.1.2.10) (Glycine cleavage system T protein)	0.996	0.497

Table A5: Genes identified exclusively by ESM as differentially expressed. Posterior inclusion probabilities, effect sizes (posterior means of β), and corresponding protein names from the UniProt database (www.uniprot.org) are also reported. For some genes, entries exist in the database, but the corresponding proteins are uncharacterized (annotated as "Uncharacterized protein").

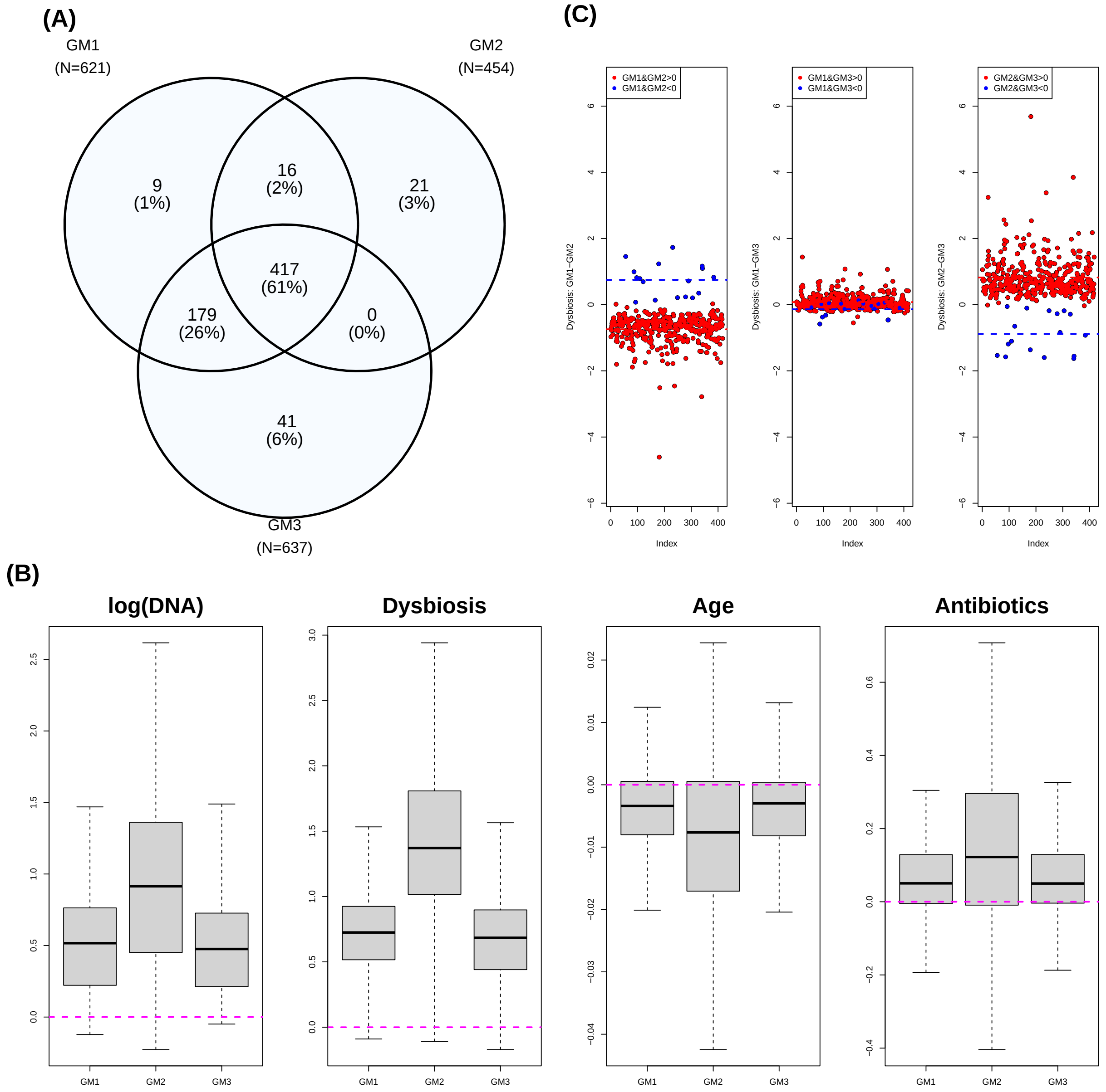

Figure A3: (A) Venn diagram of genes identified as differentially expressed with respect to the dysbiosis variable under each Gaussian model, with N denoting the number of identified genes. (B) Boxplots (with outliers omitted) of effect sizes for log(DNA), dysbiosis, age, and antibiotic use (i.e., posterior means of α, β, γ_1, and γ_2) for the 417 commonly identified genes. (C) Pairwise differences in the dysbiosis effect sizes among the common genes across models.
